# Corrigendum to Primary health care expenditure in the Americas: measuring what matters

**DOI:** 10.26633/RPSP.2022.210

**Published:** 2022-11-30

The *Pan American Journal of Public Health* draws readers’ attention to an error in the following article, pointed out by the authors.

In page 3, table 1, HC.3.4 Outpatient long-term care (health), should read HC.3.4 Home-based long-term care (health).

